# Supplementary material for: Molecular characterization of the effects of heat shock on the infection cycle progression and productivity of the baculovirus expression vector system
Source: PLoS One. 2025 Apr 2;20(4):e0320917. doi: 10.1371/journal.pone.0320917 (PMC11964234; doi:10.1371/journal.pone.0320917)
Supplement: S1 File — (PDF) [file pone.0320917.s001.pdf]

Molecular characterization of the effects of heat shock on the infection cycle progression and productivity of the Baculovirus Expression Vector System

Enrique Paz-Cortés, A. Ruth Pastor, Roberta Salinas-Marín, Octavio T. Ramírez, and Laura A. Palomares

Supplementary information 1 (S1 File)

Table 1. Primers used to amplify and sequence two inducible HSP70 genes from *Spodoptera frugiperda*

Gene	Primers
hsp70-1<br>(identified in this work as *hsp68*)	Primers for gene amplification<br>hsp70-1 FWD<br>TTATTAGTCTACTTCTTCTACGGTGGGCC CCGAGTTCGCTTG<br>hsp70-1 REV<br>TTATTAGTCTACTTCTTCTACGGTCGGTC CCGAGTTCGC<br><br>Primers for sequencing<br>hsp70-1-seqFWD1<br>GTCACAGGCTGAGATTGACAGAATG<br>hsp70-1-seqREV2<br>GCATCGTCCAGCGCTTGTTTCAC
hsp70-2<br>(identified in this work as *hsp70Ab*)	Primers for gene amplification<br>hsp70-1 FWD<br>AATGCCAGCCATTGGAATAGATCTGGGT<br>hsp70-1 REV<br>TTAATCTACCTCTTCGATGGTAGGTCC<br><br>Primers for sequencing<br>hsp70-2-seqFWD1<br>GATCATCAACGAGCCCACAGC<br>hsp70-2-seqREV2<br>GATGGTCAGGATCGACACGTC<br>hsp70-2-seqFWD3<br>AGTCGTACGTGTTCAGCGTGAAGC

Table 2. Primers sequences for genes used in real-time quantitative PCR (qPCR) for EGFP and in Reverse Transcription qPCR for *hsp70* genes and the *28s* reference gene

Gene target / amplification efficiency	sequence (5'-3')	Predicted amplification targets using PrimerBLAST [a] [b]
hsp68<br><br>(102.97%)	FWD:<br>ATCATCAACGAGCCCACAGC<br>REV:<br>GATGGTCAGGATCGACACGTC<br><br>Reference sequence:<br>PQ108613 (this work)	1. XM_035603314.2 - PREDICTED: Spodoptera frugiperda heat shock protein 68
hsp70Ab<br>**(*hsp70Ab*-group)**<br><br>(106.14%)	FWD:<br>GATCATCAACGAGCCCACAGC<br><br>REV:<br>GATGGTCAGGATCGACACGTC<br><br>Reference sequence:<br>PQ108612 (this work)	1. XM_035600951.2 PREDICTED: Spodoptera frugiperda major heat shock 70 kDa protein Ba<br>2. XM_035600948.2 PREDICTED: Spodoptera frugiperda heat shock protein 68<br>3. XM_050699539.1 PREDICTED: Spodoptera frugiperda heat shock protein 68-like<br>4. XM_050699226.1 PREDICTED: Spodoptera frugiperda heat shock protein 68-like<br>5.1 XM_050699212.1 PREDICTED: Spodoptera frugiperda heat shock protein 68-like (LOC126910613), transcript variant X4<br>5.2 XM_050699211.1 PREDICTED: Spodoptera frugiperda heat shock protein 68-like (LOC126910613), transcript variant X3<br>5.3 XM_050699210.1 PREDICTED: Spodoptera frugiperda heat shock protein 68-like (LOC126910613), transcript variant X2<br>5.4 XM_050699209.1 PREDICTED: Spodoptera frugiperda heat shock protein 68-like (LOC126910613), transcript variant X1
28s<br><br>(107.33%)	FWD:<br>GATCATCAACGAGCCCACAGC<br>REV:<br>GATGGTCAGGATCGACACGTC<br><br>Reference sequence:<br>XR_004783999.1	1. XR_004783999.2 PREDICTED: Spodoptera frugiperda large subunit ribosomal RNA<br>2. XR_007707646.1 PREDICTED: Spodoptera frugiperda large subunit ribosomal RNA<br>3. XR_004783997.2 PREDICTED: Spodoptera frugiperda large subunit ribosomal
EGFP<br><br>(97.73%)	FWD:<br>GATCATCAACGAGCCCACAGC<br>REV:<br>GATGGTCAGGATCGACACGTC<br><br>Gene present in the Dual Reporter Baculovirus AcNPV (DRBac) (Hidalgo et al., 2017)	"No target templates were found in selected database: RefSeq Representative Genome Database (Organism limited to Autographa californica multiple nucleopolyhedrovirus)" [a].<br>(Primers are specific for EGFP gene).

[a] primer-BLAST (Ye et al., 2012) analysis was performed in April 2024. For genes *hsp68*, *hsp70Ab*-group and 28s we limited the search to *Spodoptera frugiperda* records in the RefSeq mRNA database. For EGFP we limited the search to *Autographa californica* multiple nucleopolyhedrovirus (taxid:307456) and the RefSeq representative genomes database.

[b] Different entries may refer to the same gene in *Spodoptera frugiperda.*

Table 3. BLAST hits for Expressed Sequence Tag (EST) Sf2H01963-5-1, in *S. frugiperda* [a]

EST Sf2H01963-5-1 Sequence:

CAGAATTATTTGAAACGCGATACAAAGCAGACGAAGAGAGTTCGGTGCAAACCGAGGAGTTTGAGTTTACTAGACTCGCAACGC
AAGTTTATTCAACGTTATTGTTATTATTGAGTGTGAAGAGAATTAGTGAGTGAGAAGACAAACAAAATACCAGCCATTGGAATAGA
TCTGGGTACCACATACTCGTGCGTCGGCGTGTGGCAGCACGGCAACGTGGAGATCATCGCCAACGACCAGGGCAACCGCACC
ACACCATCCTATGTGGCCTTCACGGACACGGAGCGCCTCATCGGAGACGCAGCCAAGAACCAGGTCGCCCTCAACCCCAACAA
CACGGTGTTCGACGCCAAGCGACTGATCGGAAGGAAATTCGACGACCCCAAGATCCAGGCAGACATGAAACACTGGCCCTTCA
GGGTGGTCAGCGACTGCGGCAAACCGAAGATCCAAGTGGAGTTCAAGGGGGAAACGAAACGGTTCGCGCCCGAGGAGATCAG
CAGCATGGTGCTGACGAAGATGAAGGAGACGGCGGAAGCTTACCTCGGAACGACAGTACGCGACGCGGTGATCACAGTGCCG
GCGTACTTCAACGACTCTCAGCGCCAGGCCACCAAGGACGCGGGAGCCATCGCCGGGCTGAACGTGCTCAGGATCATCAACG
AGCCCACAGCCGCTGCGCTCGCGTACGGCCTCGACAAGAACCTCAAGGGCGAGAGGAACGTCCTCATCTTCGACCTCGGCGG
CGGAACGTTCGACGTGTCAATCCTGACCATCGAC

Accession: https://www.ncbi.nlm.nih.gov/nuccore/295263226

**BLAST hits[b]**				
**Accesion**	**Description [c]**	**Coverage**	**e-value**	**Identity**
PQ108612 [d] (this work)	Heat shock protein 70Ab (hsp70Ab), complete cds	80%	0	99.52%
XM_035600951.2 [d]	PREDICTED: Spodoptera frugiperda major heat shock 70 kDa protein Ba (LOC118280663), mRNA	100%	0	99.74%
XM_050699226.1	PREDICTED: Spodoptera frugiperda heat shock protein 68-like (LOC118280708), mRNA	100%	0	99.23%
XM_050699211.1	PREDICTED: Spodoptera frugiperda heat shock protein 68-like (LOC126910613), transcript variant X3, mRNA	100%	0	98.59%
XM_050699210.1	PREDICTED: Spodoptera frugiperda heat shock protein 68-like (LOC126910613), transcript variant X2, mRNA	100%	0	98.59%
XM_050699209.1	PREDICTED: Spodoptera frugiperda heat shock protein 68-like (LOC126910613), transcript variant X1, mRNA	100%	0	98.59%
XM_035600948.2	PREDICTED: Spodoptera frugiperda heat shock protein 68 (LOC118280660), mRNA	100%	0	98.33%
XM_050699212.1	PREDICTED: Spodoptera frugiperda heat shock protein 68-like (LOC126910613), transcript variant X4, mRNA	97%	0	98.30%
MN480721.1	Spodoptera frugiperda heat shock protein 70 mRNA, complete cds	80%	0	99.68%
MN735778.1	Spodoptera frugiperda heat shock protein (Hsp70C) mRNA, complete cds	80%	0	99.68%
MN735777.1	Spodoptera frugiperda heat shock protein (Hsp70B) mRNA, complete cds	80%	0	99.68%
MN735776.1	Spodoptera frugiperda heat shock protein (Hsp70A) mRNA, complete cds	80%	0	99.68%
OM223038.1	Spodoptera frugiperda heat shock protein 70-1 mRNA, complete cds	80%	0	97.94%
KT218674.1	Spodoptera frugiperda heat shock protein 70 A1 mRNA, partial cds	68%	0	100%
XM_050699537.1	PREDICTED: Spodoptera frugiperda heat shock protein 70 A1 (LOC118280707), mRNA	46%	0	99.45%
MN735779.1	Spodoptera frugiperda heat shock protein (Hsp70D) mRNA, complete cds	80%	4E-133	80.48%
XM_050699539.1	PREDICTED: Spodoptera frugiperda heat shock protein 68-like (LOC126911604), mRNA	33%	6E-132	99.62%
OM223040.1	Spodoptera frugiperda heat shock protein 70-3 mRNA, complete cds	80%	4E-128	80%
MN480719.1	Spodoptera frugiperda heat shock protein 68-like transcript variant 1 mRNA, complete cds	80%	9E-125	79.68%
MN480717.1	Spodoptera frugiperda heat shock 70 kDa protein	77%	2E-077	75.61%

	cognate 4 mRNA, complete cds			
OM223039.1	Spodoptera frugiperda heat shock protein 70-2 mRNA, complete cds	77%	2E-077	75.61%
XM_035583160.2	PREDICTED: Spodoptera frugiperda heat shock 70 kDa protein cognate 4 (LOC118268613), mRNA	77%	2E-077	75.61%
MT774206.1	Spodoptera frugiperda heat shock cognate 70 mRNA, complete cds	77%	2E-077	75.61%
MN735789.1	Spodoptera frugiperda heat-shock protein 70A gene, promoter region	16%	4E-059	100%
KT324352.1	Spodoptera frugiperda heat shock protein 70 A1 gene, promoter region and 5' UTR	6%	4E-014	97.96%

[a] EST associated with *hsp70* gene with overexpression during AcNPV baculovirus infection in *S. frugiperda* cells, from Salem et al. 2011 and Nègre et al., 2006.

[b] BLAST (Altschul et al., 1990) search was performed in April 2024, using the non redundant nucleotide database and limiting records to *Spodoptera frugiperda*.

[c] Many entries in the list are automatically predicted genes. Different entries from different studies or different sets of annotation may refer to the same gene in *Spodoptera frugiperda*.

[d] Sequences selected for primers design to *hsp70Ab*.

Table 4. BLAST hits for Expressed Sequence Tag (EST) Sf2L00008-5-1 in *S. frugiperda* [a]

EST Sf2L00008-5-1 Sequence:

CGGCCGCGTCGACCTAAGATTGACGTCACCTTCGACCTAGACGCGAACGGTATCCTGAACGTGTCAGCCAAAGAAAACAGCACC
GGGCGCAGCAAGAACATTGTGATCAAAAACGACAAGGGTCGTTTGTCACAGGCTGAGATTGACAGAATGTTATCTGAAGCCGAG
CGATACAAGGAAGAGGACGAGCGCCAGCGACAGAGGGTGTCTGCTCGCAACCAGCTGGAGTCATACATCTTCAGCGTGAAACA
AGCGCTGGACGATGCTGGCGACAAGTTGAGTGAGCAGGACAAGCAGACGGCGCGCAACGAGTGTGACGAGGCACTGAAGTGG
CTGGACAACAACACGCTCGCTGAGAAGGAGGAGTACGAGCACAGAATGAAGGAGCTCCAGAGAACGTGCTCACCCATCATGAG
CAAAATGCACGGCGCGGGAGCTGGACCCCAACAATACGGACAACAAGCGAACTCGGGGCCCACCGTAGAAGAAGTAGACTAAA
TTCCTAAATTATTCAGTGATGAGTAAAACACTCTGATCTCTAGTCCCACCAGTATCTCAATTGTAAATTGTAAATAATGTAGCTTTTA
AGTTAAATTAAGTATTTATTTTCATGTAATGTTAAGAAAAAAAATCACAATCATGTGCTGGAAAAATAAAATA

**BLAST hits [b]**				
**Accesion**	**Description [c]**	**Coverage**	**e-value**	**Identity**
PQ108613 [d] (this work)	Spodoptera frugiperda heat shock protein 68, complete cds	73%	0	99.59%
XM_035603314.2 [d]	PREDICTED: Spodoptera frugiperda heat shock protein 68 (LOC118282298), mRNA	97%	0	93.53%
MN480719.1	Spodoptera frugiperda heat shock protein 68-like transcript variant 1 mRNA, complete cds	73%	0	99.39%
OM223040.1	Spodoptera frugiperda heat shock protein 70-3 mRNA, complete cds	73%	0	97.14%
MN735779.1	Spodoptera frugiperda heat shock protein (Hsp70D) mRNA, complete cds	73%	0	96.52%

[a] EST associated with *hsp70* gene with overexpression during AcNPV baculovirus infection in *S. frugiperda* cells, from Salem et al. 2011 and Nègre et al., 2006.

[b] BLAST (Altschul et al., 1990) search was performed in April 2024, using the non redundant nucleotide database and limiting records to *Spodoptera frugiperda*.

[c] Many entries in the list are automatically predicted genes. Different entries from different studies or different sets of annotation may refer to the same gene in *Spodoptera frugiperda*.

[d] Sequences selected for primers design to *hsp68*.

**Table 5.** Annotated genes and isoforms with high similitude to *hsp70Ab* (PQ108612 - this work)					
GenBank annotations that matches *hsp70Ab* (PQ108612 - 647 aa). Best matches fall in chromosome 16 (NC_064227.1 - 8061231..8110923) of *S. frugiperda*					
**Position (Chr 16)**	**Name**	**Gene ID**	**Transcript**	**Protein (size aa)**	**BLASTN [a] coverage / identity**
8061231 - 8063587	heat shock protein 68	118280660	XM_035600948.2	XP_035456841.2 (667)	97% / 99.58%
8071727 - 8074107	major heat shock 70 kDa protein Ba	118280663	XM_035600951.2	XP_035456844.2 (678)	97% / 99.74%
8078626 - 8092513	heat shock protein 68-like isoform X1	126910613	XM_050699209.1	XP_050555166.1 (717)	97% / 99.47%
Related sequence	heat shock protein 68-like isoform X2		XM_050699210.1	XP_050555167.1 (698)	97% / 99.63%
Related sequence	heat shock protein 68-like isoform X3		XM_050699211.1	XP_050555168.1 (686)	97% / 99.58%
Related sequence	heat shock protein 68-like isoform X4		XM_050699212.1	XP_050555169.1 (678)	97% / 99.68%
Related sequence	heat shock protein 70		MN480721 [b]	QGA73357.1 (643)	96% / 99.58%
8098464 - 8110923	heat shock protein 68-like	118280708	XM_050699226.1	XP_050555183.1 (981)	97% / 99.79%
8098603 - 8098965	heat shock protein 70 A1	118280707	XM_050699537.1	XP_050555494.1 (120)	18% / 99.73%
Related sequence	heat shock protein (hsp70A)		MN735776.1 [c]	QNN88712.1 (651)	100% / 98.72%
Related sequence	heat shock protein (hsp70B)		MN735777.1 [c]	QNN88713.1 (663)	97% / 99.52%
8098971 - 8099407	heat shock protein 68-like	126911604	XM_050699539.1	XP_050555496.1 (127)	18% / 99.73%
**Other BLASTN hits for *hsp70Ab* [a]**					
	heat shock protein (hsp70C)		MN735778.1 [c]	QNN88714.1 (702)	97% / 99.58%
	heat shock protein 70-1		OM223038.1 [d]	UZT55501.1 (638)	96% / 99.14%
	heat shock protein 70 A1 gene, promoter region and 5' UTR		KT324352.1 [e]	NA	51% / 100%
	heat shock protein 70 A1 mRNA, partial cds		KT218674 [e]	ALA09394 (178)	27% / 99.81%
**BLASTP hits for Hsp70Ab protein (XDK69987) with *Drosophila melanogaster* genes [f]**					
	**Name**	**GeneID**	**GenBank Reference Sequence**	**Size (aa)**	**Coverage / Identity**
	heat shock protein 70 Ab	44920	NP_524798.2	642	100% / 82.10%
	heat shock protein 68	42852	NP_524474.1	635	100% / 80.22%
	heat shock protein 70 Bb	48582	NP_524927.2	641	100% / 81.64%
	heat shock protein 70 Ba	44921	NP_731716.1	641	100% / 81.48%
	heat shock protein	50022	NP_788663.1	641	100% /

	70 Bbb				81.48%
	heat shock protein 70 cognate 2, isoform A	41609	NP_524339.1	633	100% / 75%

[a] A BLASTN search (Altschul et al., 1990) was conducted in September 2024 using the core nucleotide BLAST database, restricted to *Spodoptera frugiperda* sequences. Only hits with over 90% identity were included, as subsequent matches fell below 80% identity.

[b] Reference: Cui, et al., 2019.

[c] Reference: Chen et al., 2020.

[d] Reference: Direct submission.

[e] Reference: Bleckmann et al., 2015.

[f] BLASTP search (Altschul et al., 1997) was conducted in February 2024 using the NCBI Protein Reference Sequences. The list was cut for hits with identity below 75%.

NA: Not Applicable.

**Table 6.** Annotated genes and isoforms with high similitude to *hsp68* (PQ108613 - this work)					
GenBank annotations for *hsp68* matches (PQ108613 - 630 aa). Best matches fall in chromosome 20 (NC_064231.1:2927112-2929323) of *S. frugiperda*					
**Position (Chr 20)**	**Name**	**Gene ID**	**Transcript**	**Protein (size aa)**	**BLASTN [a] coverage / identity**
2927112 - 2929323	heat shock protein 68	118282298	XM_035603314.2	XP_035459207.2 (631)	100% / 97.2%
Related sequence	heat shock protein 68-like transcript variant 1		MN480719.1 [b]	QGA73355 (631)	100% / 99.74%
Related sequence	heat shock protein (Hsp70D) mRNA		MN735779 [c]	QNN88715 (631)	100% / 97.47%
**Other BLASTN hits for *hsp68* [a]**					
	heat shock protein 70-3		OM223040.1 [d]	UZT55503.1 (631)	100% / 97.52%
**BLASTP hits for Hsp68 protein (XDK69988) with *Drosophila melanogaster* genes [e]**					
	**Name**	**GeneID**	**GenBank Reference Sequence**	**Size (aa)**	**Coverage / Identity**
	heat shock protein 68	42852	NP_524474.1	635	100% / 81.89%
	heat shock protein 70 Bb	48582	NP_524927.2	641	100% / 80.66%
	heat shock protein 70 Ba	44921	NP_731716.1	641	100% / 80.5%
	heat shock protein 70 Bbb	50022	NP_788663.1	641	100% / 80.5%
	heat shock protein 70 Ab	44920	NP_524798.2	642	100% / 80.06%
	heat shock protein 70 cognate 2, isoform A	41609	NP_524339.1	633	100% / 77.65%

[a] A BLASTN search (Altschul et al., 1990) was conducted in September 2024 using the core nucleotide BLAST database, restricted to *Spodoptera frugiperda* sequences. Only hits with over 90% identity were included, as subsequent matches fell below 80% identity.

[b] Reference: Cui, et al., 2019.

[c] Reference: Chen et al., 2020.

[d] Reference: Direct submission.

[e] BLASTP search (Altschul et al., 1997) was conducted in February 2024 using the NCBI Protein Reference Sequences. The list was cut for hits with identity below 75%.

S1 File references:

Altschul, Stephen F., Warren Gish, Webb Miller, Eugene W. Myers, and David J. Lipman. "Basic local alignment search tool." *Journal of Molecular Biology* 215, no. 3 (1990): 403-410. https://doi.org/10.1016/S0022-2836(05)80360-2.

Altschul, Stephen F., Thomas L. Madden, Alejandro A. Schäffer, Jinghui Zhang, Zheng Zhang, Webb Miller, and David J. Lipman. "Gapped BLAST and PSI-BLAST: a new generation of protein database search programs." *Nucleic Acids Research* 25 (1997): 3389-3402. https://doi.org/10.1093/nar/25.17.3389.

Bleckmann, Maren, Markus H.-Y. Fritz, Sabin Bhuju, Michael Jarek, Margitta Schürig, Robert Geffers, Vladimir Benes, Hüseyin Besir, and Joop van den Heuvel. "Genomic Analysis and Isolation of RNA Polymerase II Dependent Promoters from *Spodoptera frugiperda*." *PLoS One* 10, no. 8 (2015): e0132898. https://doi.org/10.1371/journal.pone.0132898.

Cui, G., Sun, R., Veeran, S., Shu, B., Yuan, H., & Zhong, G. (2019). Combined transcriptomic and proteomic analysis of harmine on *Spodoptera frugiperda* Sf9 cells to reveal the potential resistance mechanism. *Journal of Proteomics, 208*, 103573. https://doi.org/10.1016/j.jprot.2019.103573

Chen, Xien, Shankar C.R.R. Chereddy, Dhandapani Gurusamy, and Subba Reddy Palli. 2020. “Identification and Characterization of Highly Active Promoters from the Fall Armyworm, Spodoptera Frugiperda.” *Insect Biochemistry and Molecular Biology* 126:103455. https://doi.org/10.1016/J.IBMB.2020.103455.

Hidalgo, David, Enrique Paz, Laura A Palomares, and Octavio T Ramírez. 2017. “Real-Time Imaging Reveals Unique Heterogeneous Population Features in Insect Cell Cultures.” *Journal of Biotechnology* 259 (October): 56–62. https://doi.org/10.1016/j.jbiotec.2017.08.019.

Nègre, Vincent, Thierry Hôtelier, Anne-Nathalie Volkoff, Sylvie Gimenez, François Cousserans, Kazuei Mita, Xavier Sabau, et al. 2006. “SPODOBASE : An EST Database for the Lepidopteran Crop Pest Spodoptera.” *BMC Bioinformatics* 7: 322. https://doi.org/10.1186/1471-2105-7-322.

Salem, Tamer Z, Fengrui Zhang, Yan Xie, and Suzanne M Thiem. 2011. “Comprehensive Analysis of Host Gene Expression in Autographa Californica Nucleopolyhedrovirus-Infected Spodoptera Frugiperda Cells.” *Virology* 412 (1): 167–78. https://doi.org/10.1016/j.virol.2011.01.006.

Ye, Jian, George Coulouris, Irena Zaretskaya, Ioana Cutcutache, Steve Rozen, and Thomas L Madden. 2012. “Primer-BLAST: A Tool to Design Target-Specific Primers for Polymerase Chain Reaction.” *BMC Bioinformatics* 13 (1): 134. https://doi.org/10.1186/1471-2105-13-134/FIGURES/5.
