# Supplementary material for: Molecular characterization of the effects of heat shock on the infection cycle progression and productivity of the baculovirus expression vector system
Source: PLoS One. 2025 Apr 2;20(4):e0320917. doi: 10.1371/journal.pone.0320917 (PMC11964234; doi:10.1371/journal.pone.0320917)
Supplement: S2 File — (PDF) [file pone.0320917.s002.pdf]

Molecular characterization of the effects of heat shock on the infection cycle progression and productivity of the Baculovirus Expression Vector System

Enrique Paz-Cortés, A. Ruth Pastor, Roberta Salinas-Marín, Octavio T. Ramírez, and Laura A Palomares

Supplementary information 2 (S2 File)

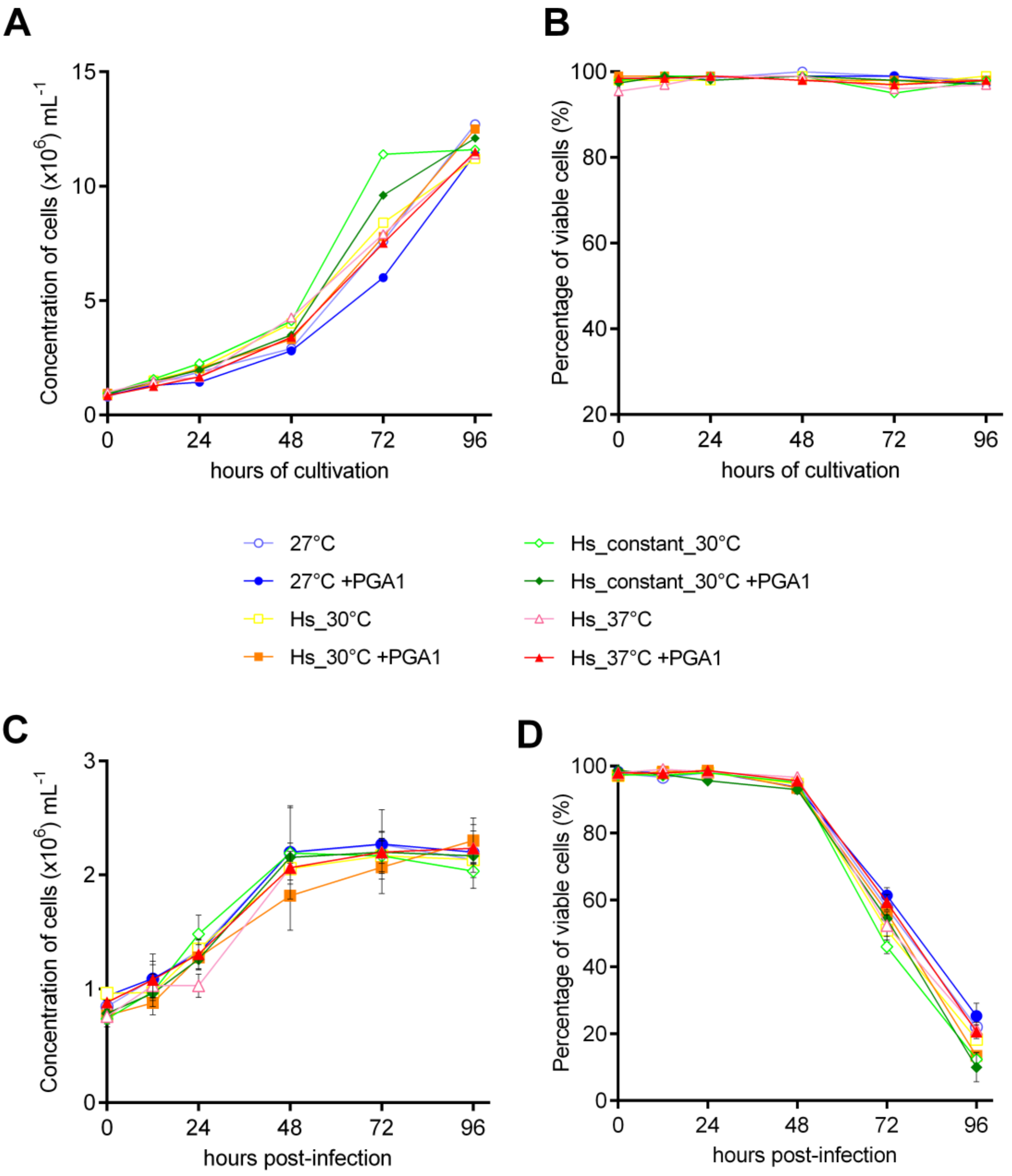

Fig 1. Dynamics of Sf9 cell concentration and viability

(**A, B**) Non-infected Sf9 cells: Cell concentration and viability, respectively, under the eight conditions combining different heat shock stimuli and Prostaglandin A1 (PGA1) supplementation. Only one replicate per condition was evaluated for non-infected cells, and no negative effects on viability or growth were observed under any heat shock condition. (**C, D**) DRBac-infected Sf9 cells: Cell concentration and viability of infected cells at a multiplicity of infection (MOI) of 1 PFU/cell. Cell concentration increased until 48 hours post-infection (hpi), followed by a decline in viability in all conditions. PGA1 treatment had a small but significant effect ($p<0.0001$) on cell viability at 72 hpi, improving viability by an average of 12±5% compared to the respective conditions without PGA1. Data reflects mean from three biological replicates and error bars represent standard deviation.

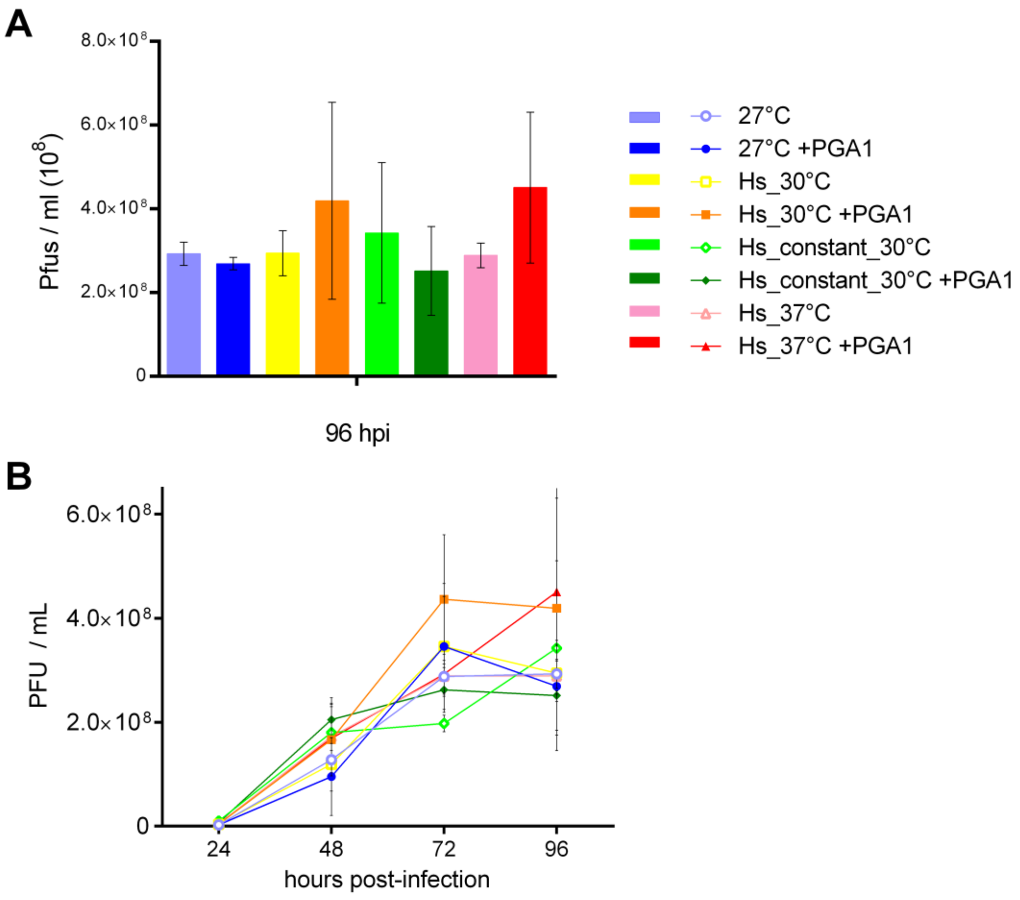

Fig 2. Production of BV progeny measured as PFU
(**A**) Plaque-forming unit (PFU) yield at 96 hours post-infection (hpi) under the eight experimental conditions. (**B**) Kinetics of PFU production (PFU/mL) from 24 to 96 hpi. Error bars represent the standard deviation from three biological replicates.

Table 1. Maximum PFU production and corresponding percentages over time

Conditions	Max PFU production (×10^8 PFU/mL)	Hours post infection 24 hpi	48 hpi	72 hpi	96 hpi
27ºC	2.93	0.9%	43.6%	98.3%	**100%**
27ºC +PGA1	3.46	0.7%	27.5%	**100%**	77.9%
Hs_30ºC	3.46	1.0%	34.4%	**100%**	85.0%
Hs_30ºC +PGA1	4.36	0.8%	38.1%	**100%**	96.1%
Hs_constant_30ºC	3.43	2.9%	52.6%	57.6%	**100%**
Hs_constant_30ºC +PGA1	2.62	2.6%	78.2%	**100%**	96.0%
Hs_37ºC	2.89	1.5%	59.5%	99.8%	**100%**
Hs_37ºC +PGA1	**4.51**	1.1%	37.4%	64.9%	**100%**

Table 2. Fold change in PFU titers across time points post-Infection (hpi)

Conditions	Fold change 24 to 48 hpi	48 to 72 hpi	72 to 96 hpi
27ºC	49.6	2.3	1.0
27ºC +PGA1	38.5	3.6	0.8
Hs_30ºC	33.1	2.9	0.9
Hs_30ºC +PGA1	45.2	2.6	1.0
Hs_constant_30ºC	18.3	1.1	1.7
Hs_constant_30ºC +PGA1	30.2	1.3	1.0
Hs_37ºC	40.3	1.7	1.0
Hs_37ºC +PGA1	35.6	1.7	1.5

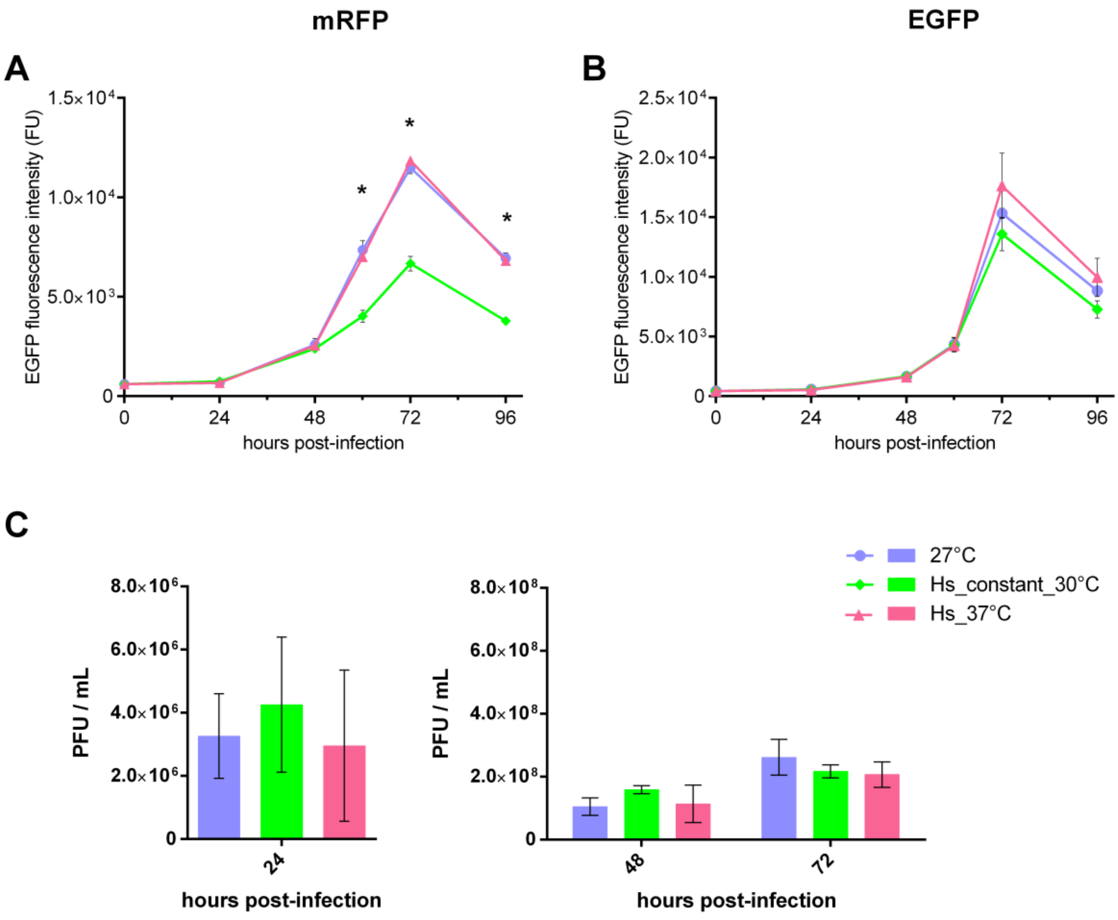

Fig 3. DRBac Replication at MOI=5: Monitoring mRFP-EGFP fluorescence and PFU output at 24, 48, and 72 hpi

(A, B) Kinetics of mRFP and EGFP fluorescence signals, respectively. **(C)** Baculovirus PFU titers (PFU/mL) at 24 hours post infection (hpi) (left, x10^6 PFU/mL), 48, and 72 hpi (right, x10^8 PFU/mL) under three conditions: control, Hs_37°C, and Hs_constant_30°C. Sf9 cells were infected with DRBac in PSFM medium at a multiplicity of infection (MOI) of 5 PFU/cell. Data are presented as mean ± standard deviation (SD) from three independent biological replicates. Asterisks indicate significant differences against the control for at least one condition at the indicated time points ($P \leq 0.05$), determined by two-factor ANOVA with Tukey's multiple comparisons.

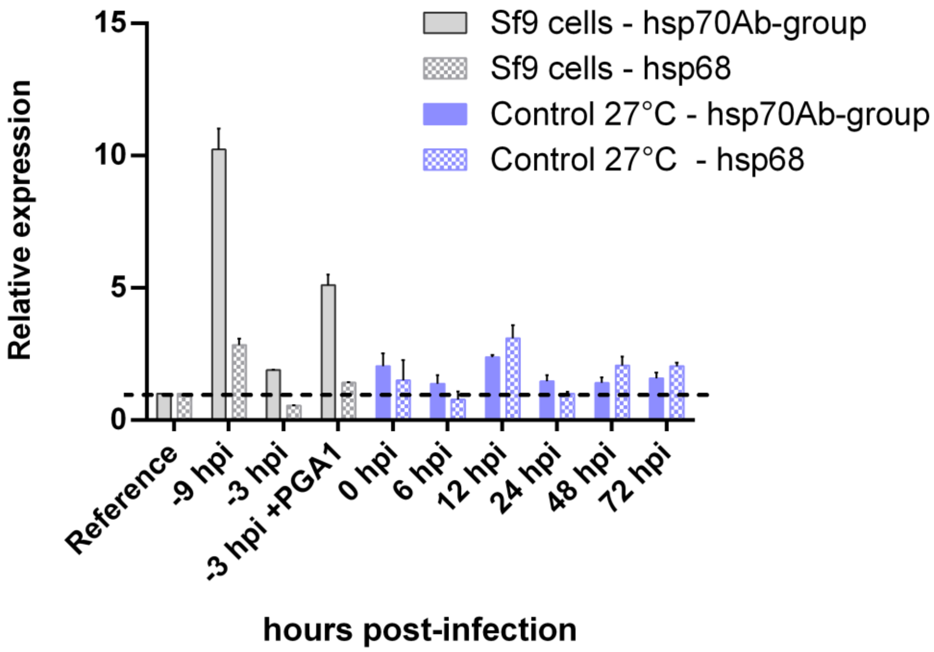

Figure 4. Relative expression of *hsp70Ab-group* and *hsp68* genes in control conditions and uninfected cells before PGA1 and heat shock treatments.

Uninfected cells (in gray) were analyzed for the expression of selected *hsp70* genes at 9 hours before infection (-9 hpi), prior to PGA1 supplementation. Additionally, expression levels were evaluated at 3 hours before infection (-3 hpi), immediately before heat shock treatments, in both normal and PGA1-supplemented conditions.

We observed a high expression of both genes at -9 hpi, with *hsp70Ab-group* showing higher expression than *hsp68*. However, both genes exhibited a continuous decline in expression, reaching their lowest levels at 6 hours post-infection (6 hpi). The PGA1 treatment resulted in increased expression of both genes at -3 hpi compared to untreated cells at the same time point, though these levels remained lower than those observed at -9 hpi. We speculate that the induction of *hsp70* gene expression prior to the assay may have been influenced by previous cell culture passages.

Dashed line indicates the expression levels of the reference sample (Sf9 uninfected cells with 72 hours of culture). 28s gene expression was used as the internal gene reference.
