# Supplementary material for: Molecular characterization of the effects of heat shock on the infection cycle progression and productivity of the baculovirus expression vector system
Source: PLoS One. 2025 Apr 2;20(4):e0320917. doi: 10.1371/journal.pone.0320917 (PMC11964234; doi:10.1371/journal.pone.0320917)
Supplement: S3 File — (PDF) [file pone.0320917.s003.pdf]

Molecular characterization of the effects of heat shock on the infection cycle progression and productivity of the Baculovirus Expression Vector System

Enrique Paz-Cortés, A. Ruth Pastor, Roberta Salinas-Marín, Octavio T. Ramírez, and Laura A Palomares

Supplementary information 3 (S3 File)

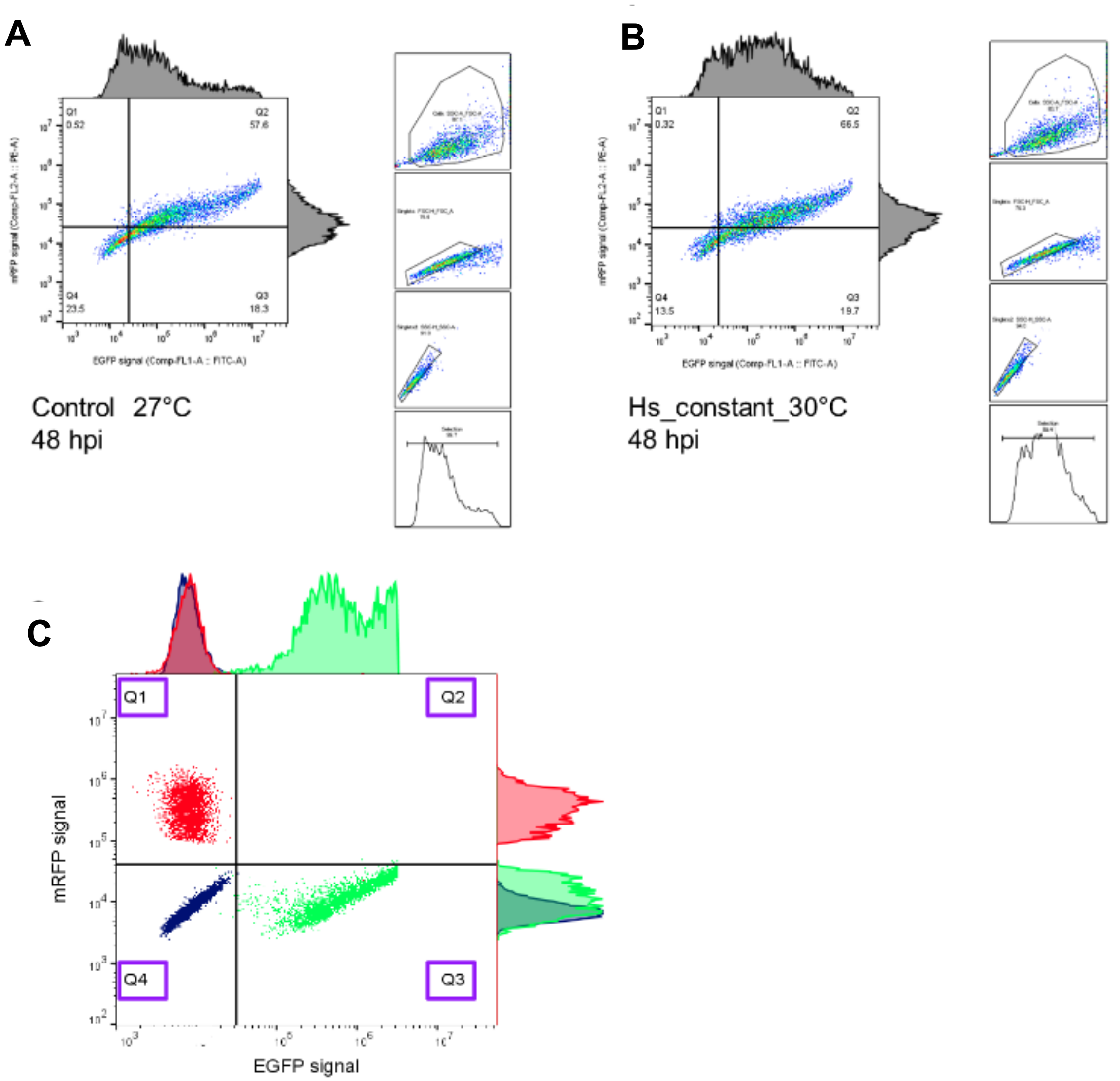

Figure 1. Example of data processing from two samples of DRBac-infected cells analyzed by flow cytometry

Panel **A** shows a dot plot of infected cells under control conditions (27°C) at 48 hours post-infection (hpi), while Panel **B** displays cells from the Hs_constant_30°C condition, also at 48 hpi. To the right of each panel are the gates used to filter data and isolate single cells, which were applied consistently across all conditions and time points. On the left side of Panels **A** and **B**, dot plots of gated single cells are shown, with cells classified into four groups based on the expression profiles of the two fluorescent reporters, mRFP (y-axis) and EGFP (x-axis). The fluorescence categories are: non-fluorescent (mRFP–/EGFP–, Q4), mRFP-positive only (mRFP+/EGFP–, Q1), EGFP-positive only (mRFP–/EGFP+, Q3), and double-positive (mRFP+/EGFP+, Q2). Histograms showing the fluorescence intensity for mRFP and EGFP are provided on the secondary X and Y axes, respectively.

Panel **C** illustrates the division of cells into quadrants (Q1 to Q4), representing the presence or absence of fluorescence signals from **mRFP** and/or **EGFP**. The X-axis corresponds to the fluorescein isothiocyanate (FITC) emission channel for EGFP, while the Y-axis represents the phycoerythrin (PE) emission channel for mRFP. Three types of control cells are visualized in the dot plots: cells infected with a baculovirus expressing only mRFP (in red, quadrant Q1), cells infected with a baculovirus expressing only EGFP (in green, quadrant Q3), and non-fluorescent cells (either infected with a baculovirus lacking fluorescent proteins or uninfected cells, shown in dark blue). Automatic compensation using FlowJo software was applied to correct spectral overlap between the reporters, ensuring accurate fluorescence measurement and distinction.

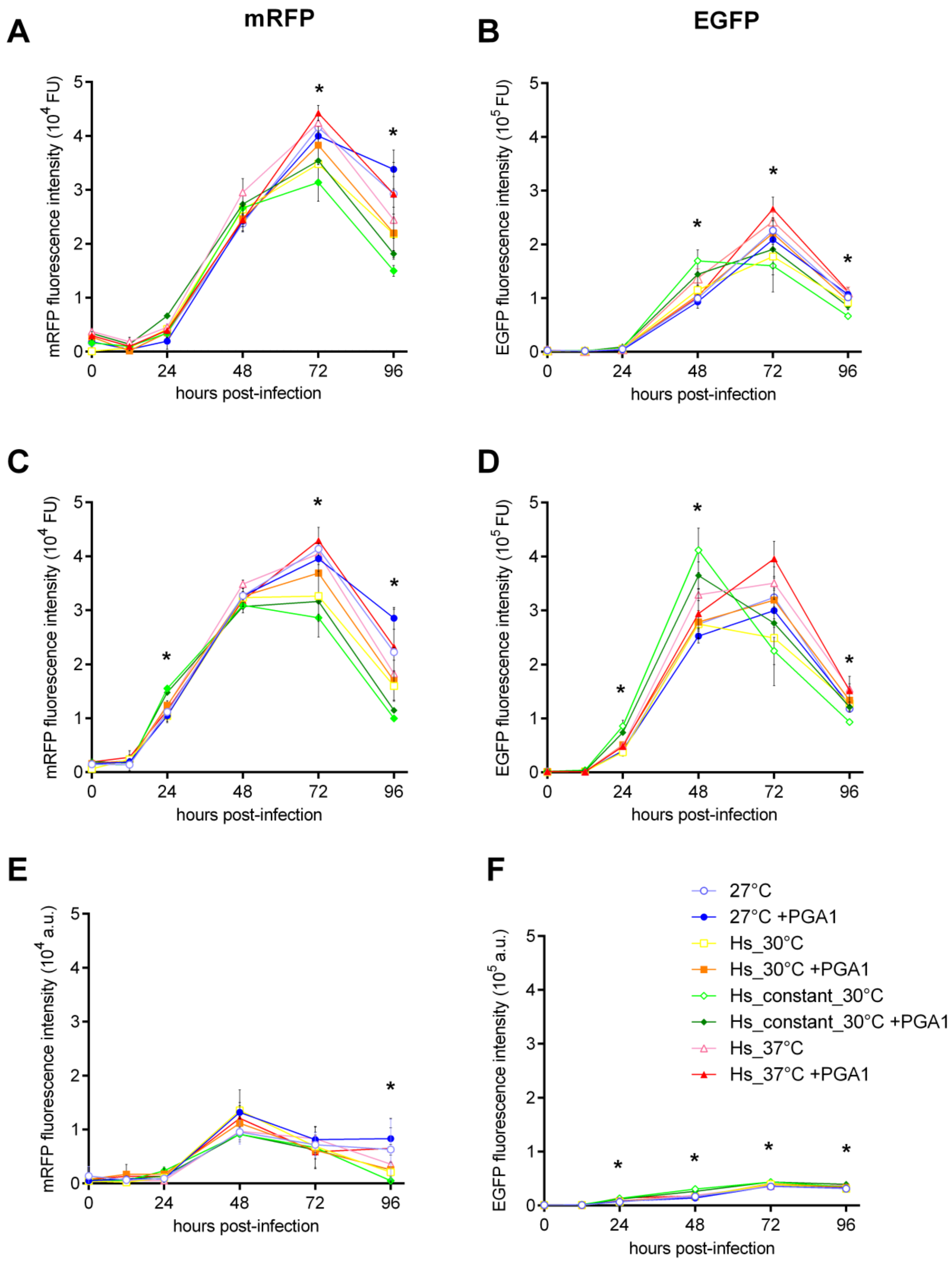

A
mRFP
B
EGFP
C
D
E
F
mRFP fluorescence intensity (10^4 FU)
EGFP fluorescence intensity (10^5 FU)
mRFP fluorescence intensity (10^4 a.u.)
EGFP fluorescence intensity (10^5 a.u.)
hours post-infection
0
24
48
72
96
27°C
27°C +PGA1
Hs_30°C
Hs_30°C +PGA1
Hs_constant_30°C
Hs_constant_30°C +PGA1
Hs_37°C
Hs_37°C +PGA1

Figure 2. Fluorescence intensity dynamics of mRFP and EGFP reporters in DRBac-infected cells via flow cytometry

Panels **A** to **F** show the mean of mRFP and EGFP fluorescence obtained by flux cytometry, from all cells or for each of the four cell subpopulations defined by their reporter expression profiles (quadrants in Figure 1, S3 File). Panels **A**, **B**, **C**, and **D** correspond to Figures 3A, 3B, 3C, and 3D in the main article, respectively, and are repeated here for easier comparison to Panels **E** and **F** in this figure.

Panels **A** and **B** depict mRFP (**A**) and EGFP (**B**) fluorescence from all cells, corresponding to an average across the four quadrants. Panels **C** and **D** highlight the mRFP and EGFP fluorescence from cells with mRFP+/EGFP+ signal (Q2, referred to as RG+ cells), which represent the largest fluorescent subpopulation, accounting for about 80% of total cells at 72 hours post-infection (hpi) (see Figure 3E in the main article), and also exhibit the highest fluorescence intensity for both mRFP and EGFP. Panel **E** shows the mRFP fluorescence for mRFP-only cells (Q1), where mRFP intensity is lower compared to the RG+ cells. In addition, these mRFP-only cells constitute less than 1% of total cells at any time or under any condition. Panel **F** displays EGFP fluorescence for EGFP-only cells (Q3), which shows considerably lower EGFP intensity compared to RG+ cells (Panel **D**).

All data were normalized by subtracting the baseline autofluorescence (0 hpi) so that non-infected cells would register near zero on both the mRFP and EGFP scales. An asterisk indicates time points in which at least one treatment condition shows a significant difference from control levels ($p < 0.05$), determined using two-factor ANOVA and Tukey's multiple comparisons of means. Detailed statistically significant differences are presented here in Table 1 S3 File.

Table 1. Statistically significant differences in mRFP and EGFP fluorescence intensity among the four cell populations, defined by their fluorescence patterns, at various hours post-infection (hpi).

		**mRFP**	**EGFP**
**Group (Quadrant)**	Time (hpi)	Condition // p-value // % relative to control	Condition // p-value // % relative to control
**All cells (Q1 to Q4)**	24		
	48		Hs_constant_30°C // **** // +70%<br>Hs_constant_30°C +PGA1 // ** // +45%<br>Hs_37°C // * // +37%
	72	Hs_30°C // * // -16 %<br>Hs_constant_30°C // **** // -24%<br>Hs_constant_30°C +PGA1 // * // -14%	Hs_30°C // ** // -21%<br>Hs_constant_30°C // **** // -28%<br>Hs_37°C +PGA1 // * // +18%
	96	Hs_30°C // ** // -25%<br>Hs_30°C +PGA1 // ** // -24%<br>Hs_constant_30°C // **** // -48%<br>Hs_constant_30°C +PGA1//****// -37%	
**mRFP & EGFP signal (Q2)**	24	Hs_constant_30°C // *** // +14%<br>Hs_constant_30°C +PGA1 //***// +12%	Hs_constant_30°C // **** // +116%<br>Hs_constant_30°C +PGA1// ***// +87%
	48		Hs_constant_30°C // *** // +49%<br>Hs_constant_30°C +PGA1// * // +32%
	72	Hs_constant_30°C // ** // -21%	
	96	Hs_constant_30°C // * // -30%<br>Hs_constant_30°C +PGA1 // *// -26%	Hs_37°C // * // +31%
**Only EGFP signal (Q3)**	24		Hs_constant_30°C // ** // +100%<br>Hs_37°C+PGA1 // ***// +112%
	48		Hs_constant_30°C // **** // +80%<br>Hs_constant_30°C+PGA1// ****//+55%
	72		Hs_30°C +PGA1 // * // +16%<br>Hs_constant_30°C // **** // +26%<br>Hs_constant_30°C+PGA1// ****//+25%

	96		Hs_constant_30°C+PGA1 // ***// +26%<br>Hs_37°C // ** // +21%
**Only mRFP signal (Q1)**	24		
	48		
	72		
	96	Hs_constant_30°C // * // -18.61%	
(p-value = * ≤ 0.05, ** ≤ 0.01 , *** ≤ .001 , **** ≤ .0001)			

Statistical analysis was conducted using the GraphPad Prism software. A two-way ANOVA followed by a Tukey's multiple comparisons test was used to assess differences between the various treatments.

Table 2. Pearson’s correlation coefficients among plaque-forming units (PFU), BV-genome copies, and mRFP or EGFP fluorescence intensities at 24, 48, and 72 hours post-infection (hpi).

**Correlation coefficients (r)**												
	**PFUs-24hpi**	**BV-genomes-24hpi**	**mRFP-24hpi**	**EGFP-24hpi**	**PFUs-48hpi**	**BV-genomes-48hpi**	**mRFP-48hpi**	**EGFP-48hpi**	**PFUs-72hpi**	**BV-genomes-72hpi**	**mRFP-72hpi**	**EGFP-72hpi**
**PFUs-24hpi**												
**BV-genomes-24hpi**	0.198											
**mRFP-24hpi**	**0.918**	0.123										
**EGFP-24hpi**	**0.959**	0.111	**0.986**									
**PFUs-48hpi**	**0.704**	0.436	**0.827**	**0.762**								
**BV-genomes-48hpi**	0.595	0.619	0.420	0.445	0.592							
**mRFP-48hpi**	-0.608	0.467	-0.554	-0.614	-0.331	-0.232						
**EGFP-48hpi**	**0.958**	0.302	**0.943**	**0.951**	**0.771**	0.541	-0.446					
**PFUs-72hpi**	**-0.716**	-0.301	-0.599	-0.647	-0.402	-0.534	0.363	**-0.772**				
**BV-genomes-72hpi**	0.285	-0.650	0.261	0.315	-0.031	-0.033	**-0.835**	0.184	-0.408			
**mRFP-72hpi**	**-0.727**	0.337	-0.676	-0.707	-0.342	-0.029	0.615	-0.665	0.330	-0.358		
**EGFP-72hpi**	-0.493	0.546	-0.424	-0.472	0.039	0.244	0.482	-0.435	0.258	-0.419	**0.911**	
**p-values (Significance levels)**												
	**PFUs-24hpi**	**BV-genomes**	**mRFP-24hpi**	**EGFP-24hpi**	**PFUs-48hpi**	**BV-genomes**	**mRFP-48hpi**	**EGFP-48hpi**	**PFUs-72hpi**	**BV-genomes**	**mRFP-72hpi**	**EGFP-72hpi**

		-24hpi				-48hpi				-72hpi		
**PFUs-24hpi**												
**BV-genomes -24hpi**	0.63839											
**mRFP-24hpi**	**0.00130**	0.77100										
**EGFP-24hpi**	**0.00016**	0.79275	**0.00001**									
**PFUs-48hpi**	0.05108	0.28045	0.01139	**0.02805**								
**BV-genomes -48hpi**	0.11987	0.10145	0.30074	0.26878	0.12169							
**mRFP-48hpi**	0.10945	0.24333	0.15413	0.10542	0.42269	0.58026						
**EGFP-48hpi**	**0.00018**	0.46794	**0.00044**	**0.00029**	**0.02525**	0.16626	0.26780					
**PFUs-72hpi**	**0.04582**	0.46944	0.11628	0.08265	0.32341	0.17311	0.37656	0.02481				
**BV-genomes -72hpi**	0.49443	0.08115	0.53167	0.44739	0.94206	0.93794	**0.00992**	0.66300	0.31536			
**mRFP-72hpi**	0.04103	0.41400	0.06595	0.05004	0.40736	0.94518	0.10476	0.07212	0.42467	0.38447		
**EGFP-72hpi**	0.21447	0.16116	0.29478	0.23725	0.92656	0.56011	0.22660	0.28178	0.53659	0.30206	**0.00165**	

Statistical analysis was conducted using the GraphPad Prism software. Pearson's correlation coefficient was used to evaluate the correlation between the distinct variables in the row/column's names. Significance is determined by p-values depicted in the second part of the table.
