# Supplementary material for: Molecular characterization of the effects of heat shock on the infection cycle progression and productivity of the baculovirus expression vector system
Source: PLoS One. 2025 Apr 2;20(4):e0320917. doi: 10.1371/journal.pone.0320917 (PMC11964234; doi:10.1371/journal.pone.0320917)
Supplement: Attachment — Submitted filename: Response to reviewers Paz et al.docx [file pone.0320917.s004.docx]

Molecular characterization of the effects of heat shock on the infection cycle progression and productivity of the baculovirus expression vector system

By Enrique Paz-Cortés et al.

Rebuttal letter

We thank the editors and reviewers for their constructive and careful manuscript revision. In this document, we present a point-by-point response to all comments. To facilitate reading, the original comments are in blue text below.

**Reviewer # 1:**

Comment 1 of 9 - Abstract: the wording used in the abstract suggests that wild type AcMNPV has been used - it should be made clear that recombinant AcMNPV expressing reported genes was used. The accepted abbreviation for the virus should be AcMNPV.

Thank you for this observation. We have clarified in the abstract that a recombinant AcMNPV expressing two reporter genes was used and ensured consistent use of the abbreviation AcMNPV throughout the manuscript

Comment 2 of 9 - Introduction: The replication cycle of AcMNPV is very much over simplified and inaccurate. The reader is led to understand that the four stages of the virus replication cycle have hard cut offs between the very early, early, late and very late stages. At several points in the text and in Fig 1, statements such as BV stops in the VL phase, BV starts in the L phase, polyhedrin protein/polh promoter is made/is active in the VL phase. There are no hard stops between the phases, BV starts being made from 15 hours and continues into the VL phase but starts to decline. The polh promoter starts about 15-18 hpi and builds through the L phase with a boost in the VL phase - recombinant proteins can be detected from about 18 hpi. The introduction should be redrafted to more accurately reflect the AcMNPV replication cycle.

We appreciate the reviewer’s comment regarding the oversimplification of the AcMNPV replication cycle in the original manuscript. We have revised the Introduction to provide a more accurate and detailed description of the replication cycle, emphasizing the continuity and overlap between the four phases.

Specifically, we have clarified that BV production begins during the late phase (approximately 15 hpi) and continues into the very late phase, although at reduced levels. Additionally, we have corrected the description of the polh promoter, noting that its activity begins around 15–18 hpi, with an increase in its expression at 24 hpi and peaks further during the very late phase. We added references to support the temporal division of the baculovirus cycle. We also modified the drawings and description in Figure 1 to remove the elements that suggest abrupt cut-offs in the baculovirus cycle. We believe these changes address the reviewer’s concerns and enhance the scientific accuracy of the manuscript.

Comment 3 of 9 - Further in the introduction, it is stated that Sf is a primary host for AcMNPV - it is not. Sf is not a natural host for AcMNPV but the virus replicates to unusually high titres in the Sf9 cell line, which is why the BEVS system uses Sf9 cells to produce high titre stocks of recombinant viruses. If you take a natural host cell, such as Tni (e.g. Hi5 cell line), then the virus does not replicate to such high titres but the polh promoter is much more active - giving generally higher recombinant protein yields, which is why many use Tni cells for recombinant protein production (but always Sf cells for virus production). So really this study has not been carried out in the 'natural' host for AcMNPV, however, the study can be justified because Sf cells are the cells used for the BEVS system - the introduction should be updated to reflect this.

We thank the reviewer for these insightful comments. We have revised the Introduction to clarify that *Spodoptera frugiperda*-derived cell lines, including Sf9, are among the most used cell lines for AcMNPV studies and are widely employed in BEVS for producing recombinant virus stocks. Additionally, we noted that *Trichoplusia ni-*derived cell lines, such as Hi5, are often preferred for recombinant protein production due to the stronger activity of the polh promoter in these cells.

We selected Sf9 cells for this study specifically because their ability to produce high viral titers aligns with our focus on investigating the effects of early HSP70 gene induction on the viral cycle. Previous studies have shown that early inhibition of HSP70 proteins significantly reduces viral DNA replication and BV production. This supports our hypothesis that earlier alterations (induction) in HSP70 protein levels may have a greater impact on viral titers and BV-genome production.

The manuscript has been updated to reflect these considerations and to provide more accurate context for the selection of Sf9 cells in this study. A reference has been added.

Comment 4 of 9 - Final point, I don't think it is 'crucial' to the development of BEVS that we know the effect of heat shock proteins, it is helpful or useful but not crucial.

We agree with your comment. We have revised the manuscript to replace the term "crucial" with "useful". This acknowledges that while understanding the effects of Hsp70 proteins is not indispensable, it provides valuable insights into the baculovirus-host cell relation, and we think our results can be useful to enhance the performance of the system.

Comment 5 of 9 - Materials and Methods: In the results, figures and text refer to PFU of virus but in the methods, TCID50 is used to titrate viruses - please state how PFU was redrived from TCID50.

We have updated the Materials and Methods section to explicitly state how PFU values were derived from TCID50. Specifically, the PFU/mL was calculated using the formula:
PFU/mL=TCID50 /mL×0.69, as described in (Mena et al., 2003). This reference also details the standard conditions and cell concentrations that we routinely use for seeding Sf9 cells in 96-well plates for tittering assays. Additionally, we have improved the information by including a mention of the Reed-Muench method, which was used to calculate the TCID50 values.

Comment 6 of 9 - Results: In 3.1, the results are described but I think a key result has been overlooked namely that the heat shock for constant 30C results in a timing difference for BV production compared to the 27 C control - at 30 C BV peaks at 48 and then declines where at 27 C, BV continues to increase until the last time point sampled at 72. It may have been helpful in this study to have gone beyond 72 h to see when the controls peaks (this may be a consequence of the authors thinking of the replication cycle in discrete phases rather than in a continuous cycle). Later results do support the initial data in 3.1 that continuous heat shock at 30 results in timing differences (e.g. Fig 3).

Thank you for your thoughtful feedback. We appreciate your observation regarding the timing differences in BV production under the constant 30°C heat shock condition compared to the 27°C control. Upon reviewing your comment and revisiting our data, we believe there may have been some misunderstanding regarding the results presented in Figure 2.

From our data, all conditions show a peak in BV production (both PFU and BV-genomes) at 72 hpi (except BV-genomes at Hs_37°C). For the constant_30°C conditions, there is still an increase in BV titer between 48 and 72 hpi, with the proportion of increase being just 1.1 and 1.3 times their levels at 48 hpi (without PGA1 and with PGA1, respectively). These proportions represent the lowest increments among the tested conditions, but they confirm a continuous increase from 48 to 72 hpi, rather than a decline as suggested in your comment. This is reflected also in the specific titers production in Table 2.

We routinely evaluate PFUs production up to 96 hpi every 24 hours, particularly in experiments conducted at MOI of 1 or lower. Extending the analysis to 96 hpi, we found only two conditions—Hs_constant_30°C and Hs_37°C +PGA1—showed further increments in PFUs (1.7- and 1.5-fold increases, respectively). However, BV-genome levels were not measured at 96 hpi due to a significant drop in cell viability (approximately 20% across conditions), which could compromise the reliability of the assay. This is why we did not present the comparison of both data at this time point. Additionally, we typically collect virus stocks before viability drops below 40% to avoid problems related to excess of cellular debris.

We acknowledge that viewing the viral replication cycle as a series of discrete phases may oversimplify its dynamics, potentially leading to inaccurate interpretations of the data. To address this, we have made revisions in the introduction, as suggested, to emphasize the continuous nature of the cycle. Additionally, we have included PFU titration data at 96 hpi as supplemental material (Fig 2 in S2 File) , recognizing the importance of this aspect highlighted by your comment. The supplemental materials now include a graphical representation of BV titration from 24 to 96 hpi, along with two tables: one showing the maximum production for each condition and the relative production proportions at each time point (Table 2 in S2 File), and another detailing the proportion of increase between consecutive sampling points (Table 3 in S2 File).

We appreciate your suggestion to extend the study beyond 72 hpi. While we have included data for PFU production at 96 hpi in this response, future experiments will aim to assess BV-genomes and other parameters at extended time points, provided cell viability remains sufficient for reliable measurements.

Comment 7 of 9 - Coexpression of two reporter genes demonstrated a clear shift in the timing of expression, to earlier in the replication cycle but I could not see clear evidence of an increase in expression overall. Therefore, the benefits of heat shock proteins may be beneficial to certain types of recombinant proteins, e.g. those that need to be processed (such as glycoproteins or secreted proteins) before the cell machinery becomes compromised through virus infection.

We appreciate your observation regarding the shift in timing of reporter gene expression and agree with your insights. This comment highlights three important aspects:

1) As you mentioned, the observed timing shift could be particularly beneficial to produce recombinant proteins that require processing (e.g., glycoproteins or secreted proteins) or are toxic to cells. Also, we think that it is worthy to keep researching the conditions that improved BV titters at earlier time points to discover elements that may be useful to improve the production of recombinant BV.

2) We also found it noteworthy that under certain heat shock conditions (e.g., Fig. 3D), EGFP expression peaked earlier. Specifically, the levels of EGFP expression at 48 hpi under the Hs_constant_30ºC condition were comparable to the maximum levels observed at 72 hpi in the control and other conditions. This earlier peak could have practical implications for bioprocesses where reducing production time is advantageous.

3) As future directions, to fully assess the potential benefits of these culture modifications, future experiments will explore the effects of the selected conditions (constant_30°C and Hs_37°C) on baculoviruses expressing a variety of recombinant proteins. This includes proteins regulated by early, late, or very late promoters, and under different MOI. Additionally, these studies may evaluate the quality of the produced proteins, including glycosylation profiles and secretion efficiency, to evaluate and determine the broader applicability of this approach.

We have updated the results section about the fluorescence analysis and the discussion section to emphasize these points and ensure their relevance and limitations are clear.

Comment 8 of 9 - Such observations have been made before and are the basis of a number of modified Sf9 cell lines stably expressing genes that aid in the expression of some proteins. I can't see this being referenced in the discussion. I think based on the results presented, the discussion and conclusion need to be modified to be more specific in the claims about the benefits of coexpressing/inducing heatshock proteins.

Thank you for your comment. We have revised the discussion section to address this point and provide a more specific and contextualized interpretation of our findings. This work represents a first approach to studying the impact that endogenous heat shock proteins and their overexpression, particularly HSP70 family proteins, may have in the baculovirus replication cycle and its productivity as a system for recombinant virus or proteins production.

While previous studies have linked these proteins to activities mainly in the early phase and replication of BV (Lyupina et al., 2010, 2011, 2014; Tung et al., 2016), others that cause recombinant Hsp70 co-expression have primarily focused on the chaperone effect of HSPs in enhancing recombinant protein folding and stability. Our findings suggest that improving *hsp70s* expression may have a broader effect, one positive effect in the baculovirus replication cycle itself (as observed through increased titers, BV-genomes, at least at 24 hpi, and accelerated production kinetics for the fluorescent reporters), while also, as a second effect, contributing to recombinant protein quality by its molecular chaperone activity, although this last was not probed in our experiments.

In the same direction, one key distinction of this proposal is the timing of HSP70 expression. It has been noted that chemical inhibition of the hsp70 induction or activity late in the baculovirus cycle, does not have the same effect as if this inhibition is done early in the infection (Lyupina et al., 2014). In a similar way, we expect that overexpression of these genes may have a bigger impact if it takes place before infection. Strategies that rely on very late promoters for HSP co-expression may have less of this effect and results more centered on the chaperone activity in the recombinant protein. In conclusion, we expect this earlier expression may provide benefits to both the viral cycle and the cellular environment supporting recombinant protein production.

There are two other aspects that may count for a difference, the first one is that inducing a stress response in the cell may implicate more genes and alterations than solely *hsp70* genes. That could have positive effects in the cell, but sustained stress can have negative effects in productivity, probably as seen in the Hs_constant_30ºC, where at 72 hpi has the lowest values for PFU and reporters' fluorescence. In comparison to cell lines with chaperones, these are usually centered in improving the processing of the recombinant protein and are based in heterologous hsp70 or other chaperone genes.

Another aspect that we believe is a very important difference is that we are centering the study on endogenous HSP70 genes and trying to differentiate the activity of at least two different inducible members of this family. There are studies in mice where they have detailed a different phenotype for the knockout of each *hsp70* gene, even when the level of similitude is high among these genes (Daugaard et al., 2007).

This brings to the table the possibility that each HSP70 gene in S. *frugiperda* may have a different role for the cell and a different effect in cases of its alteration. The same may apply to the Baculovirus and its cycle as it has been shown that these proteins are important for early events in its cycle. In this same line, we made an effort to clarify the identity and sequence of two inducible HSP70 genes (one of them with various very similar genes apparently in the same locus). This way we were able to monitor for differences in their expression in response to the infection or the combination of infection and previous heat shock. This led us to discover a higher expression by the group of *hsp70Ab*-group (in group), compared to *hsp68* (approx. 6 times to 1), but it is in *hsp68* where we found the highest and earliest changes in its expression 6 hpi.

As perspectives, thanks to the gene sequences obtained in this article, we aim to isolate and individually study the effects of the two different HSP70 genes on the baculovirus cycle and BEVS productivity. Given the sequence similarities but functional differences between HSP70 family members, we anticipate these studies will reveal specific roles for each isoform, further refining our understanding of their contributions.

In this study, we have outlined these perspectives and emphasized that while the presented findings are an initial step, they set the stage for future research. By focusing on the identity, expression timing, and optimization of endogenous HSP70 levels, we hope to provide a foundation for more targeted and effective applications of *S. frugiperda* HSP70 chaperones in the BEVS.

We have revised the discussion section to address the reviewer’s observations, incorporating additional references to provide better context for our findings and emphasizing their relevance and distinctions. We also clarified how these findings open avenues for future research, building on the foundation established by this work.

Comment 9 of 9 - The paper is very readable, and the figures well presented. Overall, once the concerns above have been addressed I think this will make a very useful contribution to those working to improve the BEVS system.

Thank you very much for your comments. Your observations have guided us in making several improvements to the article, both in terms of clarifying the presentation of the results and refining the conceptualization of the research and its relevance to the BEVS system. These changes have strengthened the clarity, relevance, and interpretation of our findings. We also hope that the revised version will contribute to the understanding and improvement of the BEVS system.

Reviewer #2

MAJOR COMMENTS

Comment 1 of 6 - Relevance of the study using a low MOI.

We understand that a low MOI infection refers to a two-step baculovirus infection process where cells are inoculated with an MOI < 1, leading to only partial infection of the cell culture. In this scenario, uninfected cells continue to proliferate, while infected cells produce recombinant protein and new virus particles, necessitating a second round of infection. This approach results in longer processing times but often achieves higher protein expression yields, as more cells are available to produce the recombinant protein.

However, most research groups typically use a high MOI (5–10) to shorten production time, utilizing a higher TOI to enhance expression. It would be valuable to understand how transferable the findings are when using a high MOI, as this aspect was not thoroughly addressed in the manuscript (it is briefly mentioned in comparisons with other labs).

Have the authors conducted any heat shock treatments with high MOI and TOI? If so, including this data in the manuscript would strengthen its applicability and understanding. I'd suggest narrowing the focus to one or two optimal conditions and performing PFU/mL and BV-genomes/mL measurements for clarity. Comparing both MOI strategies could bring an overview of the molecular characterization of the infection process.

We sincerely appreciate your thoughtful comments and suggestions. In this study, we focused on an MOI of 1 for different reasons. First, because this condition allowed us to observe more pronounced differences among the evaluated heat shock conditions. However, to address your concern about the applicability of our findings to higher MOI, we have included in the supplementary data (Fig 3 in S2 File) the results of an experiment conducted at MOI 5 under the two conditions that showed the greatest differences at MOI 1: Hs_constant_30ºC and Hs_37ºC. In this experiment, we measured fluorescence from both reporters and PFU/mL production. We observed a slight improvement in PFU production at 24 hpi, but the titers showed no difference between conditions at 48 or 72 hpi. Moreover, the fluorescent reporters showed no significant improvement in respect to the control and lower values were observed under Hs_constant_30ºC for mRFP.

The primary aim of this study was to understand how heat shock treatments influence infection dynamics, reporter expression, BV production and the expression of both inducible HSP70 identified genes, rather than focusing on maximizing recombinant protein yields. We selected an MOI of 1 because it enables the evaluation of changes in the proportion of infected cells either at the beginning of the infection, or during secondary infection. We also wanted to observe the effects on secondary infection, especially in the constant stress condition. These aspects, particularly the secondary infections, would not be possible to assess at higher MOI due to uniform and redundant infection across the population. In addition, as you mention, a higher dose may accelerate the overall infection process, giving us less time to evaluate differences in the expression of the different products and reporters monitored.

About relevance or representativity of the data, by using a MOI of 1 PFU per cell, we expected around half of the population was infected at the start of the assay (according to Palomares et al., 2005) allowing us to capture representative data. Additionally, our measured parameters (reporter fluorescence, PFUs, and BV genomes) originate exclusively from infected cells. For *hsp70* genes expression, it can be seen in Fig. 4, that the baseline expression in uninfected cells and control conditions remains relatively lower and stable, ensuring that observed differences arise solely from the administered treatments.

Using MOI 1 also aligns with our objective to monitor *hsp70* gene expression throughout the infection. Previous studies using MOI 10 (Nobiron et al., 2003; Salem et al., 2011), reported specific expression patterns: for example, Nobiron et al. observed a 3-fold increase in hsc70 transcripts peaking at 6 hpi and declining to zero at 24 hpi, while Salem et al. reported a 100-fold increase for an *hsp70* gene with a similar peak and decline. The work of Salem et al., (2011), is apparently monitoring the *hsp68* gene reported in this project (this is proposed by target identification with the qPCR primers reported, but they show mismatches with the sequence of *hsp68* and the chance to amplify other hsp70 genes, (analyzed by Primer-BLAST)). Lyupina et al. (2010) demonstrated a synergistic effect of heat shock and baculovirus infection on Hsp70 proteins induction, with modest changes (1–2-fold) at MOI 2 and larger differences (~4-fold) at MOI 10. However, as expressed in their article, their use of Western blot with a general anti-Hsp70 antibody may potentially limit the appreciation of changes on inducible Hsp70 variants because of Hsc70 constitutive proteins.

Using this information, we chose MOI 1 aiming to minimize over-induction of *hsp70* genes, which has been seen that it can impose metabolic burdens on cells and negatively affect recombinant protein production. Previous studies suggest that excessive chaperone expression can reduce productivity (Kato et al., 2005). A low viral dose allowed us to evaluate the synergistic induction of hsp70 genes, as observed in the differences among the control infection condition and the heat shock conditions evaluated, while reducing the likelihood of negative effects associated with overexpression.

We further mitigated the challenges of MOI 1 by employing more sensitive and precise measurements, such as flow cytometry, to evaluate fluorescence on a per-cell basis and distinguish infected cells from non-infected ones. By sequencing specific inducible *hsp70* genes, we identified significant expression changes across conditions, even at 72 hpi. Although we considered cell sorting to isolate infected cells, reports indicate that it can produce sorter-induced cellular stress (SICS)(Pfister et al., 2020), because of shear stress, introducing an additional variable. Also, the time required for the fluorescent reporters to accumulate enough to produce a signal distinguishable from that of uninfected cells was about 24–48 hpi, making it unable to use this technique at early times in the assay.

Regarding the timing of heat shock, we tested different periods relative to infection (24, 12, and 4–3 hours before infection, and 4 and 12 hours after infection). We observed no significant differences when applying heat shock 24 or 12 hours before infection, with similar results for treatments at 4 hours pre- or post-infection and at 12 hpi. For this study, we prioritized pre-treatment conditions to assess cellular preconditioning effects under high (37°C) and low (30°C) stress or sustained low stress (30°C constant) from the same start point in the assay. There is work from Lyupina et al., (2010) where they show the synergic effect on Hsp70 proteins induction by combining baculovirus infection and a 37ºC heat shock at different time points after infection. While heat shock post-infection may yield effects, as observed at 4 and 12 hpi, it introduces additional variables, such as direct impacts on viral components. Since Hsp70 activity is primarily linked to early infection events, we focused on pre-treatment effects.

We acknowledge that high MOI experiments could provide valuable insights into the broader applicability of our findings. However, we think such analyses require a more extensive experimental framework with room for optimization, including different recombinant proteins and promoters in addition to the MOI. Based on our results, we identified two promising conditions, Hs_37ºC and Hs_constant_30ºC, which could serve as a foundation for future research aimed at maximizing recombinant protein yield and evaluating also the quality or characteristics of the proteins under these conditions. We are especially interested in the effect on proteins that could benefit from an increase in activity from early promoters. Also, it can be possible that this approach may work only for some proteins and conditions and not as a general modification. Our aim is to study the reasons and mechanisms behind these observations to translate it to modifications of the systems with broader applicability.

We improved the justification for MOI 1 selection in the “Heat shock design and administration on cell cultures” part under the Results section and in the discussion section. We really appreciate your suggestions, we agree that while high MOI experiments represent a valuable avenue for future studies, we believe this work successfully achieves its primary objectives. It provides a comprehensive understanding of the effects of heat shock treatments on infection dynamics and BV production under the selected MOI, contributing to the broader understanding of the AcMNPV–*S. frugiperda* system and paving the way for further exploration.

Comment 2 of 6 - PFU and BV-genome per cell.

Table 2 presents PFU and BV-genomes per cell. However, as the authors noted, not all cells will be infected, making these values unreliable when quantified per cell. Expressing the units per volume would be more appropriate, ensuring consistency. The final ratio (BV-genomes to PFU) can still be provided if it refers to the same sample volume.

Thank you for your comment regarding the presentation of PFU and BV-genomes per cell in Table 2. While volumetric yields (PFU/mL and BV-genomes/mL) are provided in Figure 2, we chose to present specific yields (per cell) in Table 2 because this metric is commonly used in productivity studies. Also, we aimed to facilitate a practical and detailed comparison of production among the eight conditions, even when not all cells were initially infected.

Additionally, the BV-genomes-to-PFU ratio offers valuable insights into the efficiency of viral production under the various conditions tested, making it a key parameter in our analysis. We used per cell productivity to calculate the ratio of BV-genomes/PFU, as it normalized our data for the small variations in cell concentrations among flasks. Importantly, this ratio (BV-genomes/PFU) remains consistent regardless of whether values are derived from volumetric or per-cell yields, as both are based on the same sample volumes.

To enhance clarity, we have revised the manuscript Results section to better explain the rationale for presenting specific yields in Table 2, aiming that this information complements the volumetric data shown in Fig 2. We believe this approach improves understanding and facilitates meaningful comparisons of productivity and infection dynamics under each condition. Thank you for your thoughtful feedback, which has helped us refine the presentation of our data.

Comment 3 of 6 - vp39 and p10 promoters.
As the authors noted, there are distinct infection phases during the baculovirus lytic cycle. The absence of cells expressing only RFP suggests a narrow time window between the expression driven by the selected late and very late promoters (vp39 and p10). This overlap makes it challenging to differentiate between infection stages, particularly in an asynchronous infection. To address this, the authors may want to consider performing a high MOI infection to synchronize expression and potentially identify time points where only RFP is expressed, as higher cell density would be achieved. Otherwise, using two different reporter genes may not be necessary, as it does not provide additional information. This is evident in Figure 3, where the temporal overlap of both reporter genes is clear.
Related to the reporters, in Section 3.3, it would be helpful to address the observed discrepancy between higher PFU values and lower fluorescence levels of mRFP.

Thank you for your thoughtful comment. We recognize the importance of clarifying the temporal dynamics of the baculovirus lytic cycle and the expression of reporter genes in our study. Upon reflection, we realize we oversimplified how the baculovirus phases develop, potentially giving the impression of strict division between phases. Reviewer 1 also pointed to us that stages of the lytic cycle are not strictly sequential but instead exhibit significant overlap in gene expression. Each phase is characterized by the onset or peak of specific gene expression and events like viral DNA replication and the production of budded viruses (BV) during the late phase, or the shift toward very-late genes expression and production of occluded virus (ODV) during the very-late phase. Many genes, like vp39, classified as a late phase gene, continue to be expressed also during the subsequent very-late phase. Similarly, while *polh* is classified as very late, its expression begins earlier, with a peak during the very late phase (Grose et al., 2021).

The activity of these promoters then naturally overlaps, but the distinct expression levels and timing they drive remain valuable for studying infection dynamics and recombinant protein expression. A more precise description of our reporters’ design is that we are monitoring promoter activity under vp39 promoter, and not strictly the late phase. Our intention was not to monitor specifically the time of the late phase but rather to examine the broader dynamics and changes under these two key promoters. Using two distinct reporters allows us to capture subtle differences in promoter behavior, which are particularly evident under stress treatments.

For example, under the Hs_constant_30ºC condition, the expression peak of EGFP shifts to 48 hpi, 24 hours earlier than in other conditions (Fig. 3D). However, mRFP under the same condition or other, does not show a significant alteration in its expression at 48 hpi (Fig. 3C). This suggests that stress conditions affect very late promoter activity more significantly than late promoter activity. The overlap in promoter activity and these observed differences highlight the necessity of using two distinct reporters to detect and characterize such effects.

We acknowledge that the overlap in reporter expression complicates differentiation between late and very late stages, particularly in asynchronous infections. However, a high MOI would not necessarily resolve this issue, as the change between these phases may happen between 18-24 hpi, and detection of fluorescence relies on reporter protein accumulation, which typically requires between 24–48 hours. This delay makes it virtually impossible to distinguish between the late and very late phases using fluorescence alone. While RT-qPCR could provide finer resolution of promoter activity over time, our study was designed to assess broader dynamics across conditions rather than identifying exact phase transitions.

Regarding the discrepancy between mRFP fluorescence and BV titers noted in previous section 3.3, we initially observed no correlation under the limited set of conditions analyzed (only the three conditions that were analyzed by RT-qPCR for theirs *hsp70* genes expression: control at 27ºC, Hs_37ºC, and Hs_constant_30ºC). However, extending the analysis to include all conditions and time points (using the mean of fluorescence levels from three biological replicates for each of the eight conditions at 24, 48, and 72 hpi) revealed a strong and significant correlation between mRFP expression and BV titers specifically at 24 hpi (r = 0.92, p = 0.0013). Interestingly, we observed a similar correlation for EGFP at 24 hpi (r = 0.96, p = 0.0002). This suggests that the correlation reflects overall baculovirus activity at this stage rather than the specific expression of mRFP under the vp39 promoter. This correlation is consistent with what is appreciated in Fig. 2, that shows that both conditions under Hs_constant_30ºC, show the highest BV titers at 24 hpi, and they also exhibit significantly higher fluorescence for both reporters at the same time point (Fig. 3C, D). Noteworthy, BV-genome levels did not correlate with either reporters or BV titers at any time.

As for the presence of cells expressing only mRFP, as we mentioned, it would require a higher-frequency sampling during the first 24 hpi to to capture cells in the temporal window when vp39 is active before *polh* driven expression begins. However, the overlap in their expression and the time required for detectable fluorescence accumulation make this distinction virtually impossible using fluorescence-based methods. Under these technical circumstances we would not expect any cell expressing only mRFP, as it would mean that that cell did not get to the very late phase or the expression of EGFP under polh is defective (which may be possible). We have observed some stress conditions that appear to induce a “stalling” effect in the late phase, resulting in altered proportions of cells expressing one reporter over the other (more mRFP-only cells). While intriguing, this phenomenon requires further investigation to be fully understood. The use of our dual-reporter baculovirus (DRBac) has been proven instrumental in identifying such unexpected behaviors across various stress conditions.

In conclusion, while we acknowledge that high MOI synchronize infection, fluorescence detection limitations and the overlap of promoter activity would still preclude distinguishing between late and very late phases just by the fluorescence of mRFP reporter. Instead, our dual-reporter system provides a practical tool for assessing how stress affects in a distinct way or magnitude the dynamics of two distinct promoter activities.

We revised the manuscript and made several changes across the entire document to clarify that we were monitoring the activity under *vp39* and *polh* promoters instead of trying to monitor specific late and very late phases. Also, we made various changes in the Results section: “Flow cytometry evaluation to monitor infection progression”**,** to improve the clarity in the differences found, the utility of two reporters and that both require time to accumulate in the cells to be distinguishable. Also, we pointed out that the mentioned correlation data is in S3 File Table 2. We appreciate your insightful feedback, which has helped us to refine our study’s presentation and interpretation.

Comment 4 of 6 - Hsp70 levels.
Analysing Hsp70 levels in a heterogeneous population (<10% infected cells) may not provide a representative sample to establish clear trends. As the authors noted, it is intriguing that the expression levels are high at 0 h post-infection (likely due to the heat shock applied hours before infection) but decrease at 6 hpi before rising again at 12 hpi. While the difference is evident compared to the control (infected cells at 27°C) is significant at 12 hpi, the observed trend remains difficult to interpret. Could the authors provide additional data or reference literature to clarify this phenomenon?

Thank you for your insightful comment. We would like to clarify a potential misunderstanding regarding the infection multiplicity used in our experiments. We used an MOI of 1, not 0.1, which means we expect about half of the cell population to be infected at the start of the assay. This infection level ensures that our analysis is representative of the infected cell population. Additionally, uninfected cells exhibit low *hsp70* expression throughout the infection period, as shown in Figure 4 (gray bar and cells at 0 hpi) and we centered the analysis in the differences against the control infection condition. This consistent, low baseline allows us to attribute changes in expression levels to the evaluated conditions rather than background variability in uninfected cells.

What we found intriguing was that the hsp70 expression at 0 hpi, measured after the 2.5-hour heat shock and a 30-minute recovery period, was higher under Hs_constant_30ºC compared to Hs_37ºC. This result aligns with reports suggesting cellular stress response may increase with stress severity, but it can be impaired when stress becomes excessive (Richter et al., 2010). We suspect that 37°C may represent a stress level so high that it impairs the cellular stress response, resulting in lower *hsp70* expression than at 30°C.

The observed lower levels at 6 hpi in the control condition are close to and not significantly different to baseline levels seen in uninfected cells (gray bar in Fig. 4). In the conditions that received a heat shock, there are different patterns for each hsp70 gene, but in the case of hsp70Ab-group, for example, the three conditions analyzed have a lower level in expression at 6 hpi than at 0hpi. Levels at 6hpi are closer within the values of control conditions at 0hpi and non-infected cells at 72 hpi, which we consider base line. All conditions show a return to these levels along the experiment, we consider this is because hsp70 expression is a very dynamic system with the capacity to return to these baseline levels within hours and get an increase in several fold expression when stimulated.

As another example, we analyzed the expression of Hsp70 genes at 9 hours before infection, just before PGA1 addition to half of the cells and 6 hours before the heat shock treatments. We found that hsp70s expression levels were high at these time points before infection, probably because of culture passages. These levels continuously decreased within hours, reaching their lowest point at 0-6 hours after the infection in the control condition. Importantly, there was a subsequent increase in Hsp70 expression under the heat shock conditions, with hsp68 showing a significant rise at 6 hpi and both genes showing the highest peak by 12 hpi. This dynamic reflects a response that is likely triggered by baculovirus infection.

Comparisons with the control condition (infection at 27°C without stress treatments) further highlight the effects of different treatments. Significant differences are observed at discrete time points, which vary between the two Hsp70 genes evaluated and are detectable even at 72 hpi. We believe these late-stage differences might result from a remaining capacity of induction in these genes due to ongoing cellular stress, such as proteotoxic stress or cell lysis induction.

In contrast, other studies, such as Salem et al. (2011) and Nobiron et al. (2003), report a peak in one Hsp70 and one Hsc70 expression, respectively, at 6 hpi, followed by a sharp decline to near-zero levels by 24 hpi. This discrepancy could be due to differences in the MOI used; their studies used an MOI of 10, while we used an MOI of 1. Additionally, those studies did not use a combination of infection with heat shock stimuli, making direct comparisons difficult. However, we believe our findings at MOI 1 represent a valuable different scenario and provide useful insights into stress responses during baculovirus infection.

A higher MOI may accelerate infection dynamics and associated cellular responses, explaining the earlier peak and decay observed in their work compared to our findings at 12 hpi. Furthermore, our analysis revealed distinct expression patterns for each Hsp70 gene. Hsp68 exhibited an earlier response, with significant differences from the control detected at 6 hpi. These differences underscore the importance of analyzing each gene individually, as their regulation and response dynamics may differ significantly under the same conditions.

In summary, we believe our results provide insights into the dynamic regulation of Hsp70 genes under different stress treatments and baculovirus infection at an MOI of 1. These differences, particularly when compared to control conditions, highlight the impact of stress treatments on Hsp70 expression and the complex interplay between cellular stress responses and baculovirus infection. The observed differences in the expression patterns of *hsp68* and *hsp70Ab* further demonstrate the distinct regulatory mechanisms of these genes, emphasizing the need for individual analysis.

We made several changes to the Results section: “Identification and complete sequencing of two *hsp70* genes responsive to heat shock and AcMNPV infection”, to improve the clarity, relevance and contextualization of the differences observed in the HSP70 genes expression. Additionally, we incorporated information of HSP70 genes expression before PGA1 supplementation and before heat shock treatment in Fig 4 in supplementary S2 File. We appreciate your comment, as it allowed us to clarify and better contextualize these observations in the report.

Comment 5 of 6

Additionally, in Figure 4, panels C and D, only the relative expression levels at 24 hpi are shown. It would be valuable to include data for 12 hpi as well to confirm the effect of PGA1 addition on expression trends.

We agree that adding this time point could provide valuable insights, particularly given that our initial analysis identified 24 hpi as the most informative time for observing differences in PFU and BV-genomes and 12 hpi as the point with higher expression for both monitored hsp70 genes. Accordingly, additional experiments were performed, and samples were taken at 12 hpi. We observed that expression at 12 hpi under PGA1-supplemented conditions appears to have similar levels of expression to 24 hpi for both genes, and also similar levels among the three evaluated conditions were observed. We did not observe the same levels of induction of HSP70 genes under the Hs_constant_30ºC condition, we suspect that PGA1 may cause the induction of distinct HSP70 genes causing an unnecessary or impaired induction of specific genes as observed under the constant_30ºC condition (non-PGA1 supplemented).Expression data for 12 hpi for PGA1-supplemented conditions are now in Figure 4 to provide a more comprehensive view of the trends and confirm the early effects of PGA1 addition. This additional data will help further elucidate the dynamics of *hsp70* genes expression interplay with PGA1 treatment and cellular responses during baculovirus infection. Thank you for this thoughtful suggestion, which has helped us improve our analysis and expand the information that provides this experiment and findings.

Comment 6 of 6

Regarding the statement, “The differences observed in titers or BV-genomes among conditions at 24 hpi indicate that the variation in productivity observed is driven by factors beyond or in addition to the altered expression of the hsp70 genes analyzed”: we recognize that the regulation of the hsp70 family is highly complex and challenging to interpret. However, based on the data provided, it is not possible to demonstrate a correlation between the expression profiles under different heat shock conditions and the observed changes in productivity (measured as reporter gene expression). Both effects appear to result from the heat shock but may not necessarily influence one another directly to produce an integrated outcome.

Thank you for your detailed comment and for pointing out the complexity of interpreting the relationship between hsp70 gene expression and productivity under heat shock conditions. We acknowledge that the data presented do not establish a clear, direct correlation between these variables, and we appreciate the opportunity to clarify this aspect of our study.

We made changes in the results section and stated explicitly that our correlation analysis was exploratory, as we aimed to investigate potential trends rather than establish definitive relationships. We explored for direct correlation since it has been shown in different articles that a chemical inhibition of *hsp70* genes or proteins lead to a reduction in BV-genomes, PFU, and recombinant proteins in the BEVS (Lyupina et al., 2010, 2014; Tung et al., 2016). We were exploring the basic opposite scenario where an overexpression of these genes may correlate with positive effects on the same products.

We observed an intriguing scenario at 24 hpi, there were positive correlations between hsp70 expression levels and productivity metrics, particularly under the Hs_constant_30ºC condition, which showed the highest levels among all conditions in all markers: PFU production, fluorescence from both reporters and the expression levels of both *hsp70* genes (except BV-genomes). However, after 24 hpi, these correlations became weaker or even negative. These findings highlight a temporal disconnect between hsp70 gene expression and productivity outcomes at later stages in infection. We agree with the reviewer that the observed trends suggest that hsp70 expression and productivity may be influenced by heat shock independently, rather than through a direct cause-and-effect relationship.

To address the reviewer’s concern, we have revised the manuscript to clarify that our correlation analysis was exploratory and that the observed trends should not be interpreted as evidence of a direct interaction between hsp70 expression and productivity. Additionally, we acknowledge that further experiments, such as using larger sample sizes, different recombinant proteins, monitoring Hsp70 protein levels (rather than transcripts), and isolated overexpression of specific HSP70 genes, are needed to draw more definitive conclusions about the relation among stress and *hsp70* genes expression with baculovirus cycle dynamics and its productivity.

In summary, while our data suggest intriguing trends, particularly within 24 hpi, we agree that more comprehensive analyses are required to fully elucidate the relationship between *hsp70* gene expression and productivity under heat shock conditions. We appreciate your feedback, which has helped us refine the Results and Discussion sections by improving and clarifying the interpretation and presentation of our findings.

MINOR COMMENTS:

Comment 1 of 4 - Figure 1
Since the results are based on the infection timeline, I suggest including the hours post-infection in the figure's cycle. This addition will provide clarity on why specific time points were chosen for the time course experiments and will emphasize the differences between the primary and secondary infection cycles.

Thank you for this insightful suggestion. We agree that including the hours post-infection in Figure 1 will provide greater clarity regarding the infection timeline and the rationale for selecting specific time points for our experiments. Additionally, this will help emphasize the differences between the primary and secondary infection cycles.

This comment aligns with feedback from Reviewer 1, who noted the importance of clarifying that the transitions between phases are not abrupt and that there are overlaps in activities across these phases. To address both comments, we have revised Figure 1 to include the hours post-infection and updated the description of the infection timeline to better reflect the overlapping and dynamic nature of the phases. These changes have also been incorporated into the Introduction to provide a more accurate and functional context for the experimental findings.

Thank you again for your suggestion, which has contributed to improving the clarity and overall presentation of our study.

Comment 2 of 4 - Color Usage
While I understand that green and red are commonly used to represent EGFP and RFP, these colours are not suitable for individuals with colour blindness. I recommend considering an alternative colour scheme or employing distinct patterns to make the figure more accessible to all readers.

Thank you for mentioning this important accessibility issue. We appreciate your suggestion and have revised Figures 1 and 3, to incorporate an alternative color and pattern scheme that helps to improve accessibility for all readers.

Thank you again for bringing this to our attention and helping us improve the accessibility of our manuscript.

Comment 3 of 4 - Fig3 AND SupInf2.
The graduations on the X-axis are not evenly spaced, which is essential for this type of graph (as it is not a histogram). I recommend ensuring that the intervals are consistent to accurately represent the data and align with standard graphing conventions.

Thank you for bringing this to our attention. We have revised the X-axis graduations to ensure even spacing, providing a more accurate representation of the data and aligning with standard graphing conventions. We appreciate your observation and have updated the figure accordingly.

Comment 4 of 4 - Fig. SI_2.1 AND Fig S3.2.

The graph legend should not be presented as a separate panel. I recommend integrating it directly into the graph for better readability and a more cohesive presentation.

Thank you for this suggestion. We have made the requested changes and integrated the legend directly into the graph. We appreciate your observation, which has helped enhance the readability of the figures.

**References**

Daugaard, M., Rohde, M., & Jäättelä, M. (2007). The heat shock protein 70 family: Highly homologous proteins with overlapping and distinct functions. *FEBS Letters*, *581*(19), 3702–3710. <https://doi.org/10.1016/j.febslet.2007.05.039>

Grose, C., Putman, Z., & Esposito, D. (2021). A review of alternative promoters for optimal recombinant protein expression in baculovirus-infected insect cells. *Protein Expression and Purification*, *186*. <https://doi.org/10.1016/J.PEP.2021.105924>

Kato, T., Murata, T., Usui, T., & Park, E. Y. (2005). Improvement of the production of GFPuv-β1,3-N- acetylglucosaminyltransferase 2 fusion protein using a molecular chaperone-assisted insect-cell-based expression system. *Biotechnology and Bioengineering*, *89*, 424–433. <https://doi.org/10.1002/bit.20362>

Lyupina, Y. V., Dmitrieva, S. B., Timokhova, A. V., Beljelarskaya, S. N., Zatsepina, O. G., Evgen’ev, M. B., & Mikhailov, V. S. (2010). An important role of the heat shock response in infected cells for replication of baculoviruses. *Virology*, *406*(2), 336–341. <https://doi.org/10.1016/J.VIROL.2010.07.039>

Lyupina, Y. V., Orlova, O. V., Abaturova, S. B., Beljelarskaya, S. N., Lavrov, A. N., & Mikhailov, V. S. (2014). Egress of budded virions of Autographa californica nucleopolyhedrovirus does not require activity of Spodoptera frugiperda HSP/HSC70 chaperones. *Virus Research*, *192*, 1–5. <https://doi.org/10.1016/j.virusres.2014.08.002>

Lyupina, Y. V., Zatsepina, O. G., Timokhova, A. V., Orlova, O. V., Kostyuchenko, M. V., Beljelarskaya, S. N., Evgen’ev, M. B., & Mikhailov, V. S. (2011). New insights into the induction of the heat shock proteins in baculovirus infected insect cells. *Virology*, *421*(1), 34–41. <https://doi.org/10.1016/J.VIROL.2011.09.010>

Mena, J. A., Ramírez, O. T., & Palomares, L. A. (2003). Titration of non-occluded baculovirus using a cell viability assay. *BioTechniques*, *34*(2), 260–264. <https://doi.org/10.2144/03342bm05>

Nobiron, I., O’Reilly, D. R., & Olszewski, J. A. (2003). Autographa californica nucleopolyhedrovirus infection of Spodoptera frugiperda cells: A global analysis of host gene regulation during infection, using a differential display approach. *Journal of General Virology*, *84*(11), 3029–3039. <https://doi.org/10.1099/vir.0.19270-0>

Palomares, L. A., Estrada-Mondaca, S., & Ramírez, O. T. (2005). Principles and Applications of the Insect Cell-Baculovirus Expression Vector System. *Cell Culture Technology for Pharmaceutical and Cell-Based Therapies*, 627–693. [https://doi.org/10.1201/9780849351068-21/](https://doi.org/10.1201/9780849351068-21/PRINCIPLES-APPLICATIONS-INSECT-CELL-BACULOVIRUS-EXPRESSION-VECTOR-SYSTEM-LAURA-PALOMARES-SANDINO-ESTRADA-MONDACA-OCTAVIO-RAM)

Pfister, G., Toor, S. M., Sasidharan Nair, V., & Elkord, E. (2020). An evaluation of sorter induced cell stress (SICS) on peripheral blood mononuclear cells (PBMCs) after different sort conditions - Are your sorted cells getting SICS? *Journal of Immunological Methods*, *487*. <https://doi.org/10.1016/J.JIM.2020.112902>

Richter, K., Haslbeck, M., & Buchner, J. (2010). The Heat Shock Response: Life on the Verge of Death. *Molecular Cell*, *40*(2), 253–266. https://doi.org/10.1016/J.MOLCEL.2010.10.006/

Salem, T. Z., Zhang, F., Xie, Y., & Thiem, S. M. (2011). Comprehensive analysis of host gene expression in Autographa californica nucleopolyhedrovirus-infected Spodoptera frugiperda cells. *Virology*, *412*(1), 167–178. <https://doi.org/10.1016/j.virol.2011.01.006>

Tung, H., Wei, S.-C., Lo, H.-R., & Chao, Y.-C. (2016). Baculovirus IE2 Stimulates the Expression of Heat Shock Proteins in Insect and Mammalian Cells to Facilitate Its Proper Functioning. *PLOS ONE*, *11*(2), e0148578. <https://doi.org/10.1371/journal.pone.0148578>
